# Chest

**DOI:** 10.4103/0971-3026.45354

**Published:** 2009-02

**Authors:** Mukund D Rahalkar, Anand M Rahalkar, Deepa Divekar, Jayashree Yelgaonkar

**Affiliations:** Department of Radiology, Sahyadri Speciality Hospital, Plot No. 30-C, Erandawane, Pune - 411 004, Maharashtra, India; 1 Department of Paediatrics, Sahyadri Speciality Hospital, Plot No. 30-C, Erandawane, Pune - 411 004, Maharashtra, India

A newborn baby was admitted for respiratory distress, soon after a Caesarian section performed for acute polyhydramnios in the third trimester. The baby had not cried after birth and, at admission, was moaning, cyanosed, drowsy, limp, and not responding to stimuli; APGAR scores were 4/10, 8/10, and 7/10. The baby showed nasal flaring and sternal and substernal retraction. All neonatal reflexes were absent and there was hypotonia of all the muscles. The baby was put under an oxygen hood, but then required mechanical ventilation.

Neuro USG showed no hemorrhage in the germinal matrix or the ventricle. Chest radiographs revealed normal lungs on day 1 [Figure 1] but showed a collapsed left lung on day 2 [Figure 2].

**Figure 1:**
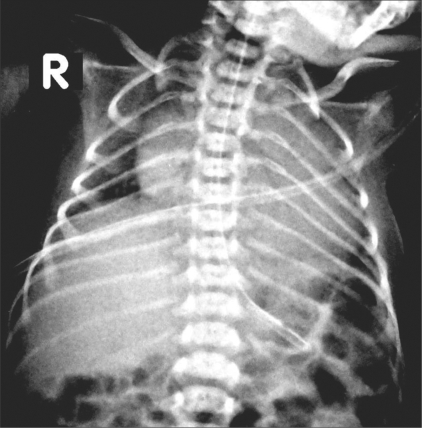
Chest radiograph on day 1

**Figure 2:**
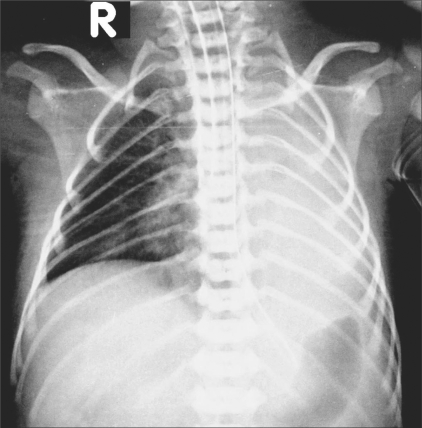
Chest radiograph on day 2. Note the left lung collapse

The baby succumbed to the respiratory distress.

## What is the diagnosis? Answer: Neuromuscular disorder

The ribs are remarkably thin on both radiographs, measuring 0.8 to 1.2 mm. in thickness. In a normal newborn, a rib usually measures about 2.5 mm in thickness.

Such thin, gracile ribs on a chest radiograph may be due to osteogenesis imperfecta, osteocraniostenosis, progeria, achondrogenesis, thin ribs-tubular bones dysmorphism, or Cockayane syndrome. All these conditions manifest at a later age (during childhood) and do not present with respiratory distress soon after birth.

The commonest cause of such signs in a newborn is a neuromuscular disorder.

Myotonic dystrophica (dystrophy) was originally considered to be a disorder confined to adult life.[1] However, during the period from 1937 to 1954, many case reports and series related to spontaneous abortions, still births, infant deaths, and hypotonia in neonates and infants showed that neuromuscular disorders could present in neonates, particularly if the mother had developed polyhydramnios during pregnancy.[1]

The first clear description of muscular dystrophy in infants / young children was given by Vanier in 1960[2] and he suggested the presence of *in utero* manifestations. He also thought that the presence of intercostal muscle involvement was supported by the finding of abnormally thin ribs on a chest radiograph.

Several subsequent case reports have mentioned this sign[3,4] (along with thin clavicles[5] as well) in neonates with various types of muscular dystrophies. It is now agreed that[3] though the sign is more likely to be seen in myotonic dystrophy, it can be seen in any neuromuscular disorder. Tall vertebrae may also occur,[6] but the neonate in our case had normal vertebrae.

The clinical differential diagnosis in this case included “floppy baby,” hypoxic-ischemic encephalopathy, and a congenital neuromuscular disorder. Ischemic encephalopathy was ruled out by neuro USG. Based on the clinical suspicion of a neuromuscular disorder and because of the presence of thin ribs on the chest radiographs, a nerve conduction velocity (NCV) test was performed on the tibialis anterior muscle, which revealed absent impulses, suggesting anterior horn cell disease of the spinal cord (spinal muscular atrophy, type I). The left lung collapse may have been either due to the respiratory disease or it may have been a complication of the mechanical ventilation.
